# Advancements in biomarkers and machine learning for predicting of bronchopulmonary dysplasia and neonatal respiratory distress syndrome in preterm infants

**DOI:** 10.3389/fped.2025.1521668

**Published:** 2025-04-25

**Authors:** Hanieh Talebi, Seyed Alireza Dastgheib, Maryam Vafapour, Reza Bahrami, Mohammad Golshan-Tafti, Mahsa Danaei, Sepideh Azizi, Amirhossein Shahbazi, Melina Pourkazemi, Maryam Yeganegi, Amirmasoud Shiri, Ali Masoudi, Heewa Rashnavadi, Hossein Neamatzadeh

**Affiliations:** ^1^ Clinical Research Development Unit, Fatemieh Hospital, Hamadan University of Medical Sciences, Hamadan, Iran; ^2^ Department of Medical Genetics, School of Medicine, Shiraz University of Medical Sciences, Shiraz, Iran; ^3^ Firoozabadi Clinical Research Development Unit, Department of Pediatrics, Iran University of Medical Sciences, Tehran, Iran; ^4^ Neonatal Research Center, Shiraz University of Medical Sciences, Shiraz, Iran; ^5^ Department of Pediatrics, Islamic Azad University of Yazd, Yazd, Iran; ^6^ Department of Obstetrics and Gynecology, School of Medicine, Iran University of Medical Sciences, Tehran, Iran; ^7^ Shahid Akbarabadi Clinical Research Development Unit, Iran University of Medical Sciences, Tehran, Iran; ^8^ School of Medicine, Ilam University of Medical Sciences, Ilam, Iran; ^9^ School of Medicine, Iran University of Medical Sciences, Tehran, Iran; ^10^ Department of Obstetrics and Gynecology, School of Medicine, Iranshahr University of Medical Sciences, Iranshahr, Iran; ^11^ School of Medicine, Shiraz University of Medical Sciences, Shiraz, Iran; ^12^ School of Medicine, Shahid Sadoughi University of Medical Sciences, Yazd, Iran; ^13^ School of Medicine, Tehran University of Medical Sciences, Tehran, Iran; ^14^ Mother and Newborn Health Research Center, Shahid Sadoughi University of Medical Sciences, Yazd, Iran

**Keywords:** biomarkers, bronchopulmonary dysplasia, neonatal respiratory distress syndrome, machine learning, predictive models, preterm infants

## Abstract

Recent advancements in biomarker identification and machine learning have significantly enhanced the prediction and diagnosis of Bronchopulmonary Dysplasia (BPD) and neonatal respiratory distress syndrome (nRDS) in preterm infants. Key predictors of BPD severity include elevated cytokines like Interleukin-6 (IL-6) and Tumor Necrosis Factor-alpha (TNF-α), as well as inflammatory markers such as the Neutrophil-to-Lymphocyte Ratio (NLR) and soluble gp130. Research into endoplasmic reticulum stress-related genes, differentially expressed genes, and ferroptosis-related genes provides valuable insights into BPD's pathophysiology. Machine learning models like XGBoost and Random Forest have identified important biomarkers, including CYYR1, GALNT14, and OLAH, improving diagnostic accuracy. Additionally, a five-gene transcriptomic signature shows promise for early identification of at-risk neonates, underscoring the significance of immune response factors in BPD. For nRDS, biomarkers such as the lecithin/sphingomyelin (L/S) ratio and oxidative stress indicators have been effectively used in innovative diagnostic methods, including attenuated total reflectance Fourier transform infrared spectroscopy (ATR-FTIR) and high-content screening for ABCA3 modulation. Machine learning algorithms like Partial Least Squares Regression (PLSR) and C5.0 have shown potential in accurately identifying critical health indicators. Furthermore, advanced feature extraction methods for analyzing neonatal cry signals offer a non-invasive means to differentiate between conditions like sepsis and nRDS. Overall, these findings emphasize the importance of combining biomarker analysis with advanced computational techniques to improve clinical decision-making and intervention strategies for managing BPD and nRDS in vulnerable preterm infants.

## 1 Introduction

Bronchopulmonary dysplasia (BPD) and neonatal respiratory distress syndrome (nRDS) are major complications in preterm infants, often resulting in long-term health issues (1, 2). Identifying reliable biomarkers for these conditions is crucial for improving clinical outcomes. Studies have highlighted potential biomarkers and mechanisms involved in their pathogenesis. Endothelin-1 (ET-1) is a promising biomarker for predicting BPD, as it is associated with bronchoconstriction and pulmonary hypertension, with elevated levels indicating early risk in preterm infants with nRDS (3–5). Interleukin-6 (IL-6) is another significant biomarker, with higher serum levels correlating with BPD development, underscoring the role of inflammation in lung injury (6). Oxidative stress is also critical, as preterm infants have immature antioxidant defenses, leading to increased lung tissue damage (7, 8). Clara cell protein expression has been investigated as a potential biomarker for lung injury and BPD risk (9). Furthermore, microbial colonization, particularly by Pneumocystis jirovecii, may exacerbate respiratory distress in preterm infants through mechanisms like surfactant disruption and mucus overproduction (10, 11). This underscores the need for biomarkers that reflect both inflammatory responses and microbial presence to effectively assess BPD risk.

Recent advancements in predicting BPD and nRDS in preterm infants utilize biomarkers and machine learning techniques to enhance early diagnosis and clinical outcomes (12, 13). Machine learning algorithms have significantly improved predictive capabilities for these conditions. Studies employing weighted gene co-expression network analysis (WGCNA) and machine learning have identified novel serum biomarkers linked to BPD, such as TMCC2, GYPA, and BPGM (14). This approach enhances prediction accuracy and integrates complex datasets, deepening understanding of factors influencing BPD and nRDS (12, 15, 16). Combining clinical data with machine learning models has shown promise in predicting outcomes for neonates with respiratory distress. Analyzing clinical signs and laboratory results through machine learning can inform predictive models, assisting clinicians in managing preterm infants and leading to personalized treatment plans that improve survival rates and reduce long-term complications associated with BPD and nRDS (17–19).

This review aims to systematically explore and synthesize existing literature on advancements in biomarkers and machine learning algorithms that improve the prediction and early identification of BPD and nRDS in preterm infants. By examining the interplay between biological markers and machine learning techniques, this review seeks to highlight innovative approaches that may enhance clinical outcomes, inform diagnostic strategies, and facilitate timely management of these prevalent neonatal conditions. Additionally, the review will identify research gaps, outline future investigation directions, and propose recommendations for integrating these technological advancements into clinical practice.

## 2 Bronchopulmonary dysplasia

### 2.1 Cytokines and inflammatory biomarkers

BPD is a significant complication in preterm infants, marked by chronic lung inflammation and injury, with cytokines and inflammatory biomarkers playing crucial roles in its development (20, 21). IL-6 is notably elevated in infants who develop BPD, with levels above 46.125 pg·ml^−1^ correlating with increased risk, supported by a high area under the curve (AUC) of 0.849 (22). Tumor Necrosis Factor-alpha (TNF-α) is also commonly elevated, disrupting lung development through inflammatory pathways. Acute inflammation is linked to Interleukin-1 (IL-1) and Interleukin-8 (IL-8), while reduced levels of the anti-inflammatory cytokine Interleukin-10 (IL-10) may predispose newborns to BPD by failing to regulate inflammation effectively (23–25). Inflammatory biomarkers such as the Neutrophil-to-Lymphocyte Ratio (NLR), soluble gp130, and 8-Hydroxy-2′-deoxyguanosine (8-OHdG) are emerging as predictors of BPD severity, with elevated NLR indicating systemic inflammation and high soluble gp130 levels reflecting inflammatory response involvement (26, 27). The inflammatory response in BPD includes immune cell infiltration, especially monocytes and neutrophils, which can further damage tissue. Additionally, the combination of hyperoxia from supplemental oxygen and inflammation exacerbates oxidative stress, contributing significantly to lung injury and BPD pathogenesis. A study by Gao et al. explored the predictive value of umbilical cord blood Interleukin-6 (UCB IL-6) in 414 preterm infants born before 32 weeks. The study found a significant correlation between UCB IL-6 levels and BPD severity, achieving an area under the receiver operating characteristic curve (AUROC) of 0.815, particularly differentiating Grade 2–3 BPD patients. Four machine learning models—XGBoost, CatBoost, LightGBM, and Random Forest—produced micro-average AUROC values of 0.841, 0.870, 0.851, and 0.878, respectively, with UCB IL-6 consistently identified as the most important feature across all models. This indicates that UCB IL-6 is a valuable biomarker for assessing the risk of severe BPD in preterm infants, aiding clinical decision-making (7).

### 2.2 Gene expression and stress-related biomarkers

#### 2.2.1 Endoplasmic reticulum stress-related genes (ERSGs)

Recent studies highlight the involvement of endoplasmic reticulum (ER) stress-related genes (ERSGs) in BPD pathogenesis. ER stress, caused by the accumulation of misfolded proteins in the ER, disrupts cellular function, which is particularly relevant for lung development in premature infants (28–30). Research shows a strong correlation between ERSG expression and various immune cells, like neutrophils and macrophages, suggesting that ER stress significantly affects immune responses in BPD patients. ERSGs are crucial for essential processes supporting alveolar development, including mitochondrial function maintenance, oxidative stress regulation, and inflammation control. Infants with BPD exhibit significant dysregulation of ERSGs compared to those without, indicating a broader role for ER stress in the disease's progression and severity (31, 32). Understanding ERSG implications may lead to novel therapeutic strategies to mitigate lung damage in infants at risk for or affected by BPD. Ziyu Tao et al. examined the relationship between ERSGs and BPD using interpretable machine learning on the GSE32472 dataset. They identified two molecular clusters linked to ER stress in BPD patients, with notable immune cell infiltration and altered ERSG expression compared to controls. Among the models tested, the support vector machine (SVM) showed superior discriminative ability, particularly when combined with five specific genes, indicating its potential for clinical risk assessment and therapeutic interventions in BPD (32). These findings enhance our understanding of BPD mechanisms and highlight biomarkers that may assist in its management, underscoring ERSGs' role in the disease's pathophysiology and their potential as predictive markers.

#### 2.2.2 Differentially expressed genes (DEGs)

Recent studies have identified differentially expressed genes (DEGs) associated with BPD, improving our understanding of its pathophysiology and potential therapeutic targets. One study found 49 cluster-specific DEGs by integrating genes related to ER stress with those linked to BPD, highlighting the complex relationship between ER stress and immune responses in the disease. Additionally, RNA sequencing of cord blood revealed 1,685 genes involved in pathways that inhibit lymphopoiesis and adaptive immune responses, with 471 DEGs significant at *p* < 0.01 and a total of 1,685 at *p* < 0.05. These DEGs were enriched in crucial biological pathways, particularly T cell receptor signaling and inflammatory responses such as IL-6/JAK/STAT3 signaling. Key genes associated with lung alveolarization and BPD development included SPARC and AGER (33). Moreover, elevated neutrophil activation and altered T cell homeostasis were observed in BPD infants, indicating a heightened inflammatory response that may contribute to disease progression. The correlation between DEGs and immune cell types, such as neutrophils and macrophages, suggests these genes could influence immune infiltration patterns in the lungs of affected infants (32, 34). The study by Luo et al. explores potential biomarkers in the peripheral blood of neonates with BPD. Utilizing the Gene Expression Omnibus dataset GSE32472, the researchers identified 470 DEGs and conducted WGCNA, revealing 1,351 significant genes. The intersection of DEGs and WGCNA modules yielded 273 common genes, which were subjected to functional enrichment analysis. To identify hub genes, the researchers applied three machine learning algorithms—SVM-RFE, LASSO, and Random Forest—resulting in the identification of CYYR1, GALNT14, and OLAH as potential BPD biomarkers. Additionally, flunisolide, budesonide, and beclomethasone were predicted as therapeutic drugs linked to these biomarkers (14). The findings indicate that CYYR1, GALNT14, and OLAH may serve as diagnostic indicators for BPD, thereby enhancing clinical diagnosis and preventive strategies while highlighting the relevance of gene expression profiles in understanding the condition.

#### 2.2.3 Ferroptosis-related genes

Ferroptosis, an iron-dependent form of cell death characterized by lipid peroxidation, has emerged as a significant factor in various respiratory diseases, such as acute respiratory distress syndrome (ARDS), asthma, and acute lung injury (35). Recent findings indicate its involvement in the pathogenesis of BPD, particularly in premature infants. Studies have shown elevated free iron levels in the umbilical cord blood of these infants, with cumulative enteral iron increasing BPD risk. Additionally, abnormal iron accumulation has been detected in the lungs of BPD models, and therapies targeting ferroptosis demonstrate potential in mitigating hyperoxia-induced lung damage, suggesting a critical role for this regulated cell death pathway in respiratory health and disease (36, 37). Recent research by Fang et al. has highlighted the potential of ferroptosis-related genes as diagnostic biomarkers for BPD. Utilizing datasets from the GEO and FerrDb databases, the study identified 23 ferroptosis-related differentially expressed mRNAs (FRDE-mRNAs) primarily involved in biological processes such as autophagy, fatty acid metabolism, and ferroptosis itself. Four hub genes—LPIN1, ACADSB, WIPI1, and SLC7A11—were selected through machine learning algorithms including LASSO, SVM-RFE, and Random Forest to construct a diagnostic nomogram, which demonstrated strong predictive performance as evidenced by satisfactory receiver operating characteristic (ROC) and calibration curves. Furthermore, the study assessed immune cell infiltration in BPD, revealing significant differences in eight immune cell markers between BPD patients and controls. This research not only elucidates the role of ferroptosis in BPD but also identifies key biomarkers that could facilitate timely diagnosis and intervention, providing a foundation for future investigations into the underlying mechanisms of the disease. Their research led to the development of a diagnostic nomogram, pinpointing the relevance of ferroptosis in BPD (38).

#### 2.2.4 Cuproptosis-related genes (CRGs)

Cuproptosis is a recently discovered mechanism of cell death distinct from traditional apoptosis, triggered by the accumulation of copper ions within cells, resulting in toxic effects. This mechanism interacts uniquely with lipid-acylated TCA cycle proteins, and the disruption of these proteins due to elevated copper levels sets off a chain of molecular events leading to cell death (39, 40). Research into Cuproptosis has opened new avenues for understanding cell death mechanisms and developing therapeutic strategies to regulate copper levels in various diseases. In the context of bronchopulmonary BPD, especially among preterm infants, Cuproptosis-Related Genes (CRGs) such as NFE2L2 and GLS have been identified as differentially expressed between affected individuals and healthy controls, with these genes located on chromosome 2 showing a positive correlation and ties to immune responses (41, 42). Through bioinformatics analyses, including the GSE108754 dataset, researchers, notably Mingxuan Jia et al., uncovered differential expression patterns of CRGs. In particular, NFE2L2 was more expressed in control samples, while GLS was prominent in treatment groups, and immune infiltration studies revealed significant differences in monocyte cell populations. Applying weighted correlation network analysis (WGCNA) identified the top 25% of gene expressions, leading to the identification of five key marker genes—NFATC3, ERMN, PLA2G4A, MTMR9LP, and LOC440700—using various machine learning algorithms like Generalized Linear Models, Random Forest, SVM, and Extreme Gradient Boosting. The GLM model showed superior accuracy across validation datasets, suggesting these genes could be promising targets for early diagnosis and targeted therapies for BPD, highlighting the significant role of novel cell death mechanisms in the disease (41).

### 2.3 Genetic biomarkers and signatures

#### 2.3.1 Genetic risk factors

Studies on heritability, including twin analyses, indicate that genetic factors contribute to 53%–79% of BPD susceptibility (43, 44), underscoring the role of familial influences. Investigations into candidate genes have revealed critical genetic variants, such as mutations in SPOCK2, which is vital for alveolarization, and surfactant-associated genes like ABCA3, linked to both nRDS and BPD development due to their impact on surfactant metabolism and lung development (45, 46). Although genome-wide association studies have struggled to consistently identify common genetic variations related to BPD, the complex genetic landscape appears to be influenced primarily by multiple rare variants (45). Understanding these genetic predispositions is crucial for identifying at-risk infants and developing prevention strategies. A study by Dai et al. sought to enhance the early prediction of BPD in premature infants by integrating genetic factors with clinical data. Analyzing a cohort of 245 infants born before 32 weeks gestation through exome sequencing, they identified 30 genes associated with BPD and 21 linked to severe BPD. Their predictive models, incorporating these genetic risk gene sets with clinical factors, achieved an AUROC of 0.915 for BPD, outperforming the clinical-only model (AUROC 0.814, *P* = 0.013), and a similar improvement was seen in the severe BPD model (AUROC 0.907 vs. 0.826, *P* = 0.016). These findings underscore the significant role of genetic factors in BPD susceptibility and indicate that the proposed predictive model may facilitate better risk stratification in premature infants (47).

#### 2.3.2 Transcriptomic gene signature

Gene expression profiling provides deep insights into cellular functionality across various tissues, particularly through whole genome microarray analysis, which is the leading method for evaluating transcriptomic variations in specific populations. Although transcriptome-based risk and prediction scores show promise for various adult diseases, their application in BPD has not been extensively explored (48). A recent study by Alvaro Moreira et al. aimed to develop a peripheral blood transcriptomic gene signature using artificial intelligence to identify preterm neonates at risk for BPD. By analyzing whole blood microarray data from 97 very low birth weight neonates on day five of life, the research defined BPD based on the necessity of positive pressure ventilation or supplemental oxygen by 28 days of age. The study found significant differences in gestational age and birth weight between neonates diagnosed with and without BPD. Out of 33,252 genes assessed, 4,523 showed a false discovery rate of less than 1%. Machine learning models that combined five genes achieved AUROC scores between 85.8% and 96.1%, demonstrating strong predictive accuracy. The identified pathways related to T cell development underscore the role of immune factors in BPD's pathogenesis. This study successfully creates a five-gene blood signature for early BPD prediction, emphasizing improved early identification and intervention strategies (49). The research utilized a rigorous methodology with careful participant assignment for model training and validation, ensuring reliability when compared to established predictors like gestational age and birth weight. Additionally, *post hoc* analyses suggest that these transcriptomic signatures retain predictive accuracy among high-risk neonates, and adherence to the TRIPOD reporting guidelines underscores the study's commitment to methodological transparency, enhancing its significance in the field.

### 2.4 Biomarkers and severities of BPD

To understand how biomarkers predict the severities of BPD it is essential to categorize the disease manifestations and examine the biomarkers linked to each. BPD can be classified into three main phenotypes based on clinical presentation: (1) Lung parenchymal disease, (2) Pulmonary vascular disease, and (3) Airway disease. While most patients exhibit significant overlap among these phenotypes, biomarkers can effectively identify the primary severities of the condition, each reflecting unique underlying mechanisms and inflammatory profiles.

1. Lung Parenchymal Disease: This phenotype is characterized by impaired alveolar development and dysregulated inflammation. Biomarkers like IL-6 are elevated in infants with lung parenchymal disease and correlate with lung injury severity. Levels of IL-6 above 46.125 pg·ml^−1^ indicate an increased risk of lung parenchymal disease, supported by an AUC of 0.849. The NLR predicts systemic inflammation, while soluble gp130 indicates inflammatory response involvement. Dysregulation of ERSGs also signifies broader roles in disease progression. Understanding these biomarkers can help identify at-risk infants and guide targeted interventions.
2. Pulmonary Vascular Disease: This phenotype features abnormal pulmonary vascular development and increased resistance. Inflammatory cytokines like TNF-α disrupt normal lung development, affecting vascular remodeling. Biomarkers linked to the vascular endothelium, such as angiopoietins and VEGF, provide insights into this phenotype, with elevated levels correlating with altered vascular development and increased risk of pulmonary hypertension in preterm infants. Additionally, ferroptosis-related genes indicate that rising iron levels contribute to oxidative stress and vascular injury. Recognizing these biomarkers can enhance our understanding and therapeutic strategies for pulmonary vascular disease in BPD.
3. Airway Disease: This phenotype involves airway inflammation, hyperreactivity, and obstructive pathology and is often associated with altered immune responses and infiltration of specific immune cells, such as neutrophils and macrophages. DEGs related to inflammatory pathways, particularly those in T cell receptor signaling and IL-6/JAK/STAT3 signaling, are vital for predicting airway disease. Elevated IL-8 and reduced IL-10 levels may predispose infants to inflammation and hyperreactivity. Recent studies have identified biomarkers like CYYR1, GALNT14, and OLAH as potential diagnostic indicators for airway disease, linked to inflammatory processes and immune cell infiltration. Understanding these biomarkers can facilitate early identification and intervention, ultimately improving clinical outcomes for affected infants.

## 3 Neonatal respiratory distress syndrome

### 3.1 Lecithin/sphingomyelin ratio (L/S ratio) biomarkers

nRDS is closely associated with fetal lung maturity, which can be evaluated through the lecithin/sphingomyelin (L/S) ratio in amniotic fluid (50, 51). This ratio is a key indicator of surfactant production, vital for lung function after birth, as lecithin is a primary component of pulmonary surfactant produced by type II pneumocytes (52). A higher L/S ratio indicates more surfactant and a reduced risk of RDS, with a ratio of 2.4 or higher suggesting mature lungs and low RDS risk. Conversely, a ratio below 1.5 indicates immature lungs and significantly increases RDS risk, while transitional lung maturity is reflected by ratios between 1.5 and 1.9, where some infants may still develop RDS but often recover. The L/S ratio is determined via amniocentesis and thin-layer chromatography, and for at-risk pregnancies, corticosteroids like betamethasone may be given to enhance surfactant production (53, 54). A study by Ahmed et al. introduced a novel diagnostic method that combines attenuated total reflectance Fourier transform infrared spectroscopy (ATR-FTIR) with machine learning to address the lack of an effective point-of-care (POC) diagnostic tool for nRDS. The diagnosis relies on the L/S ratio, with a critical threshold below 2.2 indicating nRDS. Researchers recorded ATR-FTIR spectra for L/S ratios from 1.0–3.4 using purified reagents, and employed principal component regression (PCR) and partial least squares regression (PLSR) to calibrate predictive models based on 155 raw baselined and second derivative spectra. They validated the models using an additional 104 spectra. The best-performing model was a three-factor PLSR model using second derivative spectra, achieving an *R*² of 0.967 and a mean square error (MSE) of 0.014. Predicted L/S ratios ranged from 1.0–3.4, with a prediction interval of +0.29 to −0.37 and a mean interval around the critical ratio of 2.2. The study also examined the effectiveness of mid-infrared (IR) spectroscopy for diagnosing nRDS in preterm infants by measuring key biomarkers, lecithin (L) and sphingomyelin (S). A lung lipid model containing five surfactant lipids was used to train machine learning models for predicting lipid concentrations and the L/S ratio. Advanced statistical techniques, including jackknife and bootstrap methods, quantified prediction uncertainty, indicating the L/S ratio can be determined with approximately ±0.3 mol/mol uncertainty. The study identified five significant wavenumbers related to the machine learning models, improving nRDS diagnostics. These findings highlight the potential of integrating ATR-FTIR with machine learning to develop a reliable POC device for detecting and quantifying various biomarkers using distinct mid-infrared spectral signatures, advancing real-time monitoring and outcomes in neonatal care (55).

### 3.2 Oxidative stress biomarkers genetic polymorphisms

Research into the relationship between single-nucleotide polymorphisms (SNPs) in antioxidant enzymes and oxidative stress markers in neonates is crucial, particularly related to nRDS. Oxidative stress, resulting from an imbalance between reactive oxygen species (ROS) and antioxidant defenses, is notably heightened in preterm infants, making them more vulnerable to RDS. Key antioxidant enzymes like superoxide dismutase (SOD) and catalase (CAT) play essential roles in reducing oxidative damage. Recent studies have shown that preterm neonates with RDS often exhibit increased levels of oxidative stress markers, such as malondialdehyde (MDA) and hydrogen peroxide (H2O2), alongside decreased SOD and CAT activity. Elevated advanced oxidation protein products (AOPPs) and signals of oxidative DNA damage have been correlated with more severe RDS cases, indicating a strong link between oxidative stress and disease severity (56, 57). SNPs within genes encoding these antioxidant enzymes may affect their activity and expression, influencing a neonate's ability to manage oxidative stress, with specific genetic variations potentially leading to higher stress levels and worse outcomes. Understanding these genetic factors could aid in identifying at-risk neonates and guide targeted interventions, such as antioxidant therapy, while also serving as a prognostic tool for predicting complications related to oxidative stress in this population (58). A study by Sridharan et al. explored the use of machine learning algorithms to predict oxidative stress biomarkers and SNPs associated with RDS and significant alterations in liver functions. The study found that the C5.0 algorithm had the best predictive capability for liver function alterations, achieving an AUC of 0.63, with catalase being a critical predictor. Conversely, the Bayesian network was most effective for predicting RDS, with an AUC of 0.6 and ENOS1 identified as the key predictor (59).

### 3.3 ABCA3 protein modulation

The modulation of the ABCA3 protein is crucial in the development of nRDS, particularly in preterm infants (60). As a member of the ATP-binding cassette transporter family, ABCA3 plays an essential role in pulmonary surfactant metabolism, which helps to reduce alveolar surface tension and prevent lung collapse. Mutations in the ABCA3 gene can lead to surfactant deficiencies, significantly influencing the risk of RDS; studies indicate that 14.3% of RDS infants have these mutations compared to only 3.7% of non-RDS infants. While severe forms of neonatal RDS are more commonly associated with homozygous or compound heterozygous mutations, single mutations are more frequent and account for approximately 10.9% of the attributable risk for RDS in term and late preterm infants. Identifying ABCA3 mutations may serve as a critical marker for recognizing neonates at risk for serious respiratory complications, emphasizing the need for genetic testing to inform clinical management, including considerations for extracorporeal membrane oxygenation (ECMO) interventions (61). Moreover, ABCA3 mutations have been linked to chronic interstitial lung disease later in life, suggesting that early genetic screening could have significant long-term benefits for pediatric health by differentiating genetic causes of neonatal respiratory failure from other etiologies, thereby guiding tailored therapeutic approaches (62). In a recent study by Maria Forstner et al., researchers explored high-content screening to discover pharmacologic modulators for ABCA3 deficiency, a condition linked to respiratory distress syndrome in newborns and interstitial lung disease in children. Given the lack of curative options apart from lung transplantation, the study aimed to develop a cell-based assay powered by machine learning to identify morphological differences between ABCA3 wild-type and mutant cells. Screening 1,280 FDA-approved small molecules led to the discovery of cyclosporin A as a novel and effective corrector for several ABCA3 variants, although not all. Functional assays further supported these findings, indicating that cyclosporin A could be a promising candidate for orphan drug evaluation and may facilitate controlled repurposing trials for patients with ABCA3-related diseases, addressing a significant gap in therapeutic strategies (63).

### 3.4 Crying as a biomarker

Crying in neonates, particularly preterm infants, has emerged as a valuable biomarker for assessing conditions like nRDS (64). Research has shown a strong correlation between the acoustic characteristics of an infant's cry, particularly the fundamental frequency (F0), and their physiological state, with higher F0 levels indicating greater distress affected by the autonomic nervous system's reaction to stress. Recent studies using multimodal data collection techniques—including cry analysis, electroencephalography (EEG), and near-infrared spectroscopy (NIRS)—have demonstrated that crying can accurately reflect an infant's emotional and physical condition, achieving up to 93% accuracy in distress classification. Machine learning methods have also been leveraged to differentiate between cries related to RDS and those linked to other conditions, such as sepsis, with impressive accuracy rates of 95.3%. This emphasizes the potential for developing automated diagnostic tools that rely on cry analysis (65). Understanding the nuances of cry acoustics could transform clinical practice by providing a non-invasive method to monitor preterm infant health and enable earlier interventions for RDS symptoms. Moreover, the Newborn Cry Diagnostic System (NCDS) utilizes machine learning to assess newborn cries for various health conditions, and recent advancements by Zahra Khalilzad et al. have focused on distinguishing between sepsis and RDS. Their research employed advanced machine learning techniques, particularly Multilayer Perceptron (MLP) and SVM, to analyze cry signals through musical features like the Harmonic Ratio (HR) and speech processing via Gammatone Frequency Cepstral Coefficients (GFCCs). By integrating features from both approaches and refining hyperparameters, they improved diagnostic performance, with SVM achieving a 95.3% accuracy, surpassing the MLP's accuracy of 92.49%, and underscoring the system's capability for effective class separation. This work illustrates the promise of combining diverse feature extraction methods and neural network classifiers to advance cry signal analysis and lays the groundwork for future research involving larger datasets and a wider range of pathologies (66).

### 3.5 Biomarkers and phenotypes of nRDS

To better understand nRDS and its diverse phenotypes, it is essential to explore how specific biomarkers can predict the condition's various manifestations. nRDS is not a uniform diagnosis; it represents a spectrum of clinical presentations influenced by factors such as gestational age, genetic predispositions, and environmental conditions.

1. L/S Ratio and Phenotype Correlation: The L/S ratio is a key biomarker for assessing fetal lung maturity, with predictive capabilities beyond risk classification for nRDS. Different nRDS phenotypes—mild, moderate, or severe—may correlate with varying L/S ratios. Infants with ratios near the critical threshold of 2.2 often exhibit milder symptoms and a higher chance of spontaneous recovery, while those with ratios below 1.5 are at greater risk for severe manifestations requiring intensive support. Future studies should stratify nRDS phenotypes based on L/S ratios and correlate them with clinical outcomes to enable tailored management strategies.
2. Oxidative Stress Biomarkers and Clinical Severity: Further exploration of oxidative stress biomarkers, such as MDA and H2O2, is needed to predict nRDS severity. Elevated levels of these markers may indicate increased oxidative stress and serve as predictors of severe respiratory distress. Establishing thresholds for these biomarkers alongside genetic polymorphisms related to antioxidant enzyme activity could help clinicians identify infants at risk for severe nRDS, allowing for proactive interventions. Additionally, examining the interplay between oxidative stress and inflammatory responses could shed light on their roles in different nRDS presentations.
3. ABCA3 Mutations and Long-term Outcomes: Identifying ABCA3 mutations as a risk marker for nRDS underscores the importance of genetic screening in predicting both immediate respiratory issues and long-term pulmonary health. Different mutations can result in varying degrees of surfactant dysfunction, leading to distinct nRDS phenotypes. For instance, severe mutations may cause early respiratory failure, while milder mutations could result in a gradual onset of symptoms. Understanding the specific ABCA3 mutations linked to various nRDS phenotypes can inform therapeutic approaches and help families anticipate potential long-term outcomes, including chronic lung disease risks.
4. Crying as a Multifaceted Biomarker: Analyzing the acoustic characteristics of crying may provide insights into the physiological and emotional states of neonates, potentially offering predictive value for different nRDS phenotypes. Research could differentiate cry characteristics associated with mild vs. severe nRDS and how these differ from cries linked to other neonatal conditions, like sepsis. Utilizing machine learning algorithms to analyze cry features in larger, diverse populations could lead to a robust framework for using crying as a diagnostic tool, accounting for the nuances of different nRDS manifestations.
5. Integrative Approaches to Biomarker Prediction: A comprehensive approach that integrates multiple biomarkers—such as the L/S ratio, oxidative stress markers, ABCA3 genetic variations, and acoustic cry analysis—could significantly enhance predictive accuracy for various nRDS phenotypes. Machine learning models incorporating data from these sources may uncover complex interactions and improve risk stratification. Future research should validate these integrative models in clinical settings, potentially leading to a multi-faceted diagnostic tool for real-time risk assessment and individualized treatment plans for neonates at risk of nRDS.

## 4 Discussion

Our review of biomarkers and machine learning approaches for predicting BPD and nRDS revealed a range of promising methodologies and results. Table 1 summarizes key findings in BPD prediction, highlighting the strong predictive capabilities of cytokines and inflammatory biomarkers. Machine learning models such as XGBoost and Random Forest achieved micro-average AUROC values between 0.841 and 0.878, with UCB IL-6 recognized as a critical feature. ERSGs genes demonstrated superior discriminative abilities when analyzed with SVM and specific genes. DEGs analysis identified hub genes via SVM-RFE and LASSO techniques, showcasing how advanced computational methods can reveal significant biological markers. A diagnostic nomogram based on ferroptosis-related genes showed strong ROC and calibration curves, while a genetic biomarker signature achieved AUROC values of 0.915 for general BPD and 0.907 for severe cases, surpassing traditional clinical models. In Table 2, focused on nRDS, the (L/S ratio, modeled with PLSR, produced excellent results (*R*² of 0.967). The analysis of oxidative stress biomarkers demonstrated the effectiveness of the C5.0 algorithm for liver function changes and highlighted the Bayesian network's role in RDS prediction. Innovations like high-content screening for cyclosporin A targeting ABCA3 variants show the potential of machine learning in drug discovery. The differentiation of cries associated with RDS through SVM and MLP methods underscores the diverse applications of these technologies in clinical settings.

**Table 1:** Comprehensive overview of biomarkers and machine learning approaches in predicting BPD.

Biomarker	Details	Machine learning results
Cytokines and Inflammatory Biomarkers	BPD is characterized by chronic lung inflammation with elevated IL-6 levels correlating with increased risk (AUC: 0.849). TNF-α also elevated; acute inflammation linked to IL-1 and IL-8; reduced IL-10 may predispose infants to BPD.	4 models (XGBoost, CatBoost, LightGBM, Random Forest) with micro-average AUROC values: 0.841, 0.870, 0.851, 0.878 respectively. UCB IL-6 identified as most important feature across models.
Endoplasmic Reticulum Stress-Related Genes	ER stress contributes to BPD pathogenesis by disrupting cellular function. Dysregulation of ERSGs noted in BPD infants compared to controls. Understanding these genes might lead to novel therapeutic strategies.	SVM showed superior discriminative ability when combined with five specific genes in assessing clinical risks.
Differentially Expressed Genes (DEGs)	49 cluster-specific DEGs identified; RNA sequencing revealed 471 DEGs (*p* < 0.01), enriched in pathways like IL-6/JAK/STAT3 signaling. Genes SPARC and AGER are linked to lung alveolarization and BPD development.	Identified hub genes (CYYR1, GALNT14, OLAH) using SVM-RFE, LASSO, and Random Forest from WGCNA analysis.
Ferroptosis-Related Genes	Ferroptosis implicated in BPD; elevated free iron and abnormal iron accumulation observed. Key biomarkers identified could facilitate diagnosis and intervention.	Constructed a diagnostic nomogram with four hub genes (LPIN1, ACADSB, WIPI1, SLC7A11) showing strong predictive performance (ROC and calibration curves).
Cuproptosis-Related Genes (CRGs)	Differential expression patterns of CRGs observed; genes associated with immune responses. Identified five key marker genes (NFATC3, ERMN, PLA2G4A, MTMR9LP, LOC440700) through WGCNA and various machine learning algorithms.	GLM model showed superior accuracy across validation datasets for early diagnosis and targeted therapies in BPD.
Genetic Biomarkers and Signatures	Genetic factors contribute to 53%–79% of BPD susceptibility; mutations in SPOCK2 and genes like ABCA3 identified. A study integrated genetic factors with clinical data to enhance prediction of BPD in preterm infants.	AUROC of 0.915 for BPD; AUROC of 0.907 for severe BPD, outperforming clinical-only models (*P* = 0.013 for BPD; *P* = 0.016 for severe BPD).
Transcriptomic Gene Signature	Developed a five-gene blood signature for early prediction of BPD; significant differences found in gestational age and birth weight.	AUC scores between 85.8% and 96.1% for predictive accuracy.

**Table 2:** Comprehensive overview of biomarkers and machine learning approaches in predicting nRDS.

Biomarker	Details	Machine learning results
Lecithin/Sphingomyelin Ratio (L/S Ratio)	- Key indicator of fetal lung maturity and surfactant production. - Higher ratios (≥2.4) indicate lower RDS risk; lower ratios (<1.5) indicate a higher risk. Transitional maturity is reflected in ratios of 1.5–1.9. - Determined by amniocentesis and thin-layer chromatography. - Corticosteroids (e.g., betamethasone) may be administered in at-risk pregnancies.	ATR-FTIR spectra predicted L/S ratios with a three-factor PLSR model, *R*² of 0.967, MSE of 0.014.
Oxidative Stress Biomarkers (OSBs)	- SNPs in antioxidant enzymes (e.g., SOD, CAT) are linked to oxidative stress, heightened in preterm infants vulnerable to RDS. - Preterm neonates with RDS exhibit increased oxidative stress markers (e.g., MDA, H2O2) and decreased antioxidant activity. - Elevated AOPPs and oxidative DNA damage correlate with severe RDS.	C5.0 algorithm had the best predictive capability for liver function alterations (AUC 0.63). Bayesian network most effective for RDS (AUC 0.6).
ABCA3 Protein Modulation	- ABCA3 protein is essential for pulmonary surfactant metabolism. - Mutations in the ABCA3 gene lead to an increased risk of RDS; ∼14.3% of RDS infants carry mutations. - Genetic testing for ABCA3 mutations can identify at-risk neonates and inform clinical decisions.	High-content screening identified cyclosporin A as a potential corrector for ABCA3 variants.
Crying	- Neonatal crying correlates with physiological states and distress; higher fundamental frequency (F0) levels indicate greater distress. - Multimodal data, including cry analysis and EEG/NIRS, improved distress classification accuracy (up to 93%).	SVM achieved 95.3% accuracy in differentiating cries related to RDS and other conditions; MLP achieved 92.49% accuracy.

Recent research has improved our understanding of biomarkers and risk factors for BPD in preterm infants, especially those born before 32 weeks gestation. UCB IL-6 stands out as a promising biomarker with an AUROC of 0.815, strongly correlating with BPD severity. Machine learning models, including XGBoost, CatBoost, LightGBM, and Random Forest, further refined assessments, yielding AUROC values of 0.841–0.878 while consistently identifying UCB IL-6 as the most significant feature. Investigations into ERSGs revealed distinct molecular clusters with notable immune cell infiltration and dysregulated ER stress gene expression. Machine learning algorithms identified potential biomarkers like hub genes CYYR1, GALNT14, and OLAH, as well as therapeutic options such as flunisolide and budesonide (14). An evaluation of ferroptosis identified 23 differentially expressed mRNAs related to fatty acid metabolism, with hub genes LPIN1, ACADSB, WIPI1, and SLC7A11 contributing to a diagnostic nomogram (38). Whole exome sequencing provided insights into genetic risk gene sets tied to BPD and severe BPD (sBPD), achieving AUROC values of 0.915 and 0.907, respectively (47). Additionally, analysis of cuproptosis-related genes revealed differential expression patterns, particularly increased GLS levels in affected groups (41). Through WGCNA and various machine learning algorithms, promising predictive markers like NFATC3, ERMN, PLA2G4A, MTMR9LP, and LOC440700 were identified. These studies highlight the complex pathophysiology of BPD and the essential role of immunological, genetic, and cellular mechanisms in improving early diagnosis and therapy for this vulnerable population (41).

Recent advancements in diagnosing nRDS have emphasized integrating technologies like attenuated total reflectance ATR-FTIR and machine learning, along with innovative cry-based diagnostics and genetic screening. The combination of ATR-FTIR and machine learning has shown exceptional effectiveness in predicting the L/S ratio—a key biomarker for nRDS—achieving high correlation metrics with an *R*² of 0.967, positioning it as a promising point-of-care tool for timely interventions (55). Furthermore, research into oxidative stress biomarkers and single-nucleotide polymorphisms through various machine learning approaches highlights the complexities of neonatal conditions, revealing predictive capabilities linked to genetic factors affecting respiratory and liver health (59). The innovative analysis of cry signals with advanced classifiers suggests vocalizations may serve as potential biomarkers, reflecting the multifaceted nature of neonatal diagnostics and the need for comprehensive screening strategies (66).

## 5 Clinical implications

The advancements in machine learning applications and biomarker identification for BPD and nRDS have profound clinical implications for improving outcomes in at-risk preterm infants. The detection of umbilical cord blood IL-6 as a significant biomarker, with an AUROC of 0.815, equips clinicians with a reliable tool for early diagnosis and risk stratification, especially for infants born before 32 weeks gestation. Utilizing machine learning models such as XGBoost, CatBoost, LightGBM, and Random Forest enhances predictive accuracy, paving the way for personalized interventions and better monitoring of high-risk patients. Furthermore, insights into ERSGs and potential therapeutic targets pave the way for tailored treatment strategies. In nRDS, the integration of attenuated total reflectance ATR-FTIR spectroscopy with machine learning to predict the L/S ratio represents a significant leap in point-of-care diagnostics, enabling timely interventions that can enhance respiratory outcomes. The exploration of oxidative stress biomarkers alongside genetic variations emphasizes the importance of personalized medicine in managing neonatal respiratory conditions. Additionally, investigating cry signals as potential biomarkers introduces an innovative perspective to neonatal assessments, showing that auditory cues may enhance diagnostic practices. These developments underscore the need for ongoing research to seamlessly integrate advanced methodologies into routine clinical practice, ultimately improving screening, early diagnosis, and intervention strategies for vulnerable neonatal populations and enhancing long-term health outcomes.

## 6 Limitations of current research

Despite the promising advancements in identifying biomarkers and employing machine learning techniques for diagnosing BPD and nRDS, several limitations persist. Bronchopulmonary dysplasia (BPD) is a multifaceted condition influenced by various antenatal and postnatal factors in preterm infants, which complicates the establishment of clear causal relationships in studies. The complexity of biological pathways involved in BPD and nRDS raises concerns about the robustness of identified biomarkers, as variations in gene expression and external factors like maternal health, environmental conditions, and neonatal care can confound results. While the integration of machine learning with biomarkers has enhanced the precise prediction of risk for BPD, these models likely did not establish causal inference, which must be acknowledged as a limitation of the study. Machine learning models, while demonstrating strong predictive capabilities, face challenges related to interpretability and the risk of overfitting, particularly when trained on small or homogeneous datasets. The use of umbilical cord blood levels of biomarkers such as IL-6 may also be influenced by intra- and inter-individual variability, further questioning the generalizability of findings. Additionally, the incorporation of molecular data from ERSGs and cuproptosis-related analyses presents challenges in standardizing protocols and interpreting the clinical relevance of hub genes. Ethical and logistical concerns regarding data privacy and access arise with the integration of genetic information from whole exome sequencing, potentially hindering widespread application in clinical settings. Finally, while innovative diagnostic methods like ATR-FTIR and cry-based diagnostics hold promise for early detection, their implementation may be constrained by resource availability, training requirements for healthcare personnel, and the need for validation in broader, heterogeneous clinical cohorts. These factors highlight the necessity for ongoing research and validation to address current limitations and ensure the effective translation of these advanced techniques into clinical practice for improved outcomes in vulnerable neonatal populations.

## 7 Conclusion

The review underscores the transformative potential of machine learning and advanced diagnostic techniques in predicting and understanding BPD and nRDS in preterm infants. The identification of robust biomarkers, including umbilical cord blood levels of IL-6 and various genetic markers, supports a multifaceted approach that integrates immunological, genetic, and cellular mechanisms. Machine learning models, such as XGBoost, CatBoost, LightGBM, and Random Forest, have shown substantial improvements in predictive accuracy, as indicated by their AUROC values, highlighting their clinical applicability. The investigation of ER stress-related genes and promising results from analyses of ferroptosis and cuproptosis-related genes further reveal the complexity of BPD pathophysiology, suggesting new therapeutic interventions. The use of advanced technologies like ATR-FTIR for L/S ratio prediction, along with cry-based diagnostics and high-content screening, represents a shift toward precision medicine in neonatal care. These advancements enhance understanding, early diagnosis, and treatment strategies for neonatal conditions, leading to improved clinical outcomes. The collective research indicates a significant move towards innovative methodologies, emphasizing the importance of interdisciplinary approaches that leverage machine learning and biomarker discovery.
